# X-ray near-field speckle: implementation and critical analysis

**DOI:** 10.1107/S0909049511037149

**Published:** 2011-10-05

**Authors:** Xinhui Lu, S. G. J. Mochrie, S. Narayanan, A. R. Sandy, M. Sprung

**Affiliations:** a Department of Physics, Yale University, New Haven, CT 06511, USA; b Department of Applied Physics, Yale University, New Haven, CT 06511, USA; c Advanced Photon Source, Argonne National Laboratory, Argonne, IL 60439, USA

**Keywords:** X-ray, near field, speckle, spectroscopy, scattering

## Abstract

The newly introduced coherence-based technique of X-ray near-field speckle (XNFS) has been implemented at 8-ID-I at the Advanced Photon Source. In the near-field regime of high-brilliance synchrotron X-rays scattered from a sample of interest, it turns out that, when the scattered radiation and the main beam both impinge upon an X-ray area detector, the measured intensity shows low-contrast speckles, resulting from interference between the incident and scattered beams. A micrometer-resolution XNFS detector with a high numerical aperture microscope objective has been built and its capability for studying static structures and dynamics at longer length scales than traditional far-field X-ray scattering techniques is demonstrated. Specifically, the dynamics of dilute silica and polystyrene colloidal samples are characterized. This study reveals certain limitations of the XNFS technique, especially in the characterization of static structures, which is discussed.

## Abstract 2

A coherence-based X-ray near-field speckle detector has been implemented and characterized for its capability of studying static structure and dynamics.

## 1. Introduction

Although small-angle X-ray scattering (SAXS) and X-ray photon correlation spectroscopy (XPCS) have succeeded in exploring the structure and dynamics of many interesting systems, the length scale of the observable systems is generally limited to a range from several nanometers to 100 nm, corresponding to a wavevector range of 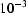
 Å^−1^ to 0.1 Å^−1^ (an angular range of 0.1° to 10°) (Dierker *et al.*, 1995 ▶; Mochrie *et al.*, 1997 ▶; Pontoni *et al.*, 2003 ▶; Falus *et al.*, 2004 ▶; Narayanan *et al.*, 2006 ▶; Lu *et al.*, 2008 ▶). Special difficulties are encountered when exploring the lower limit of the angular range, since to isolate the weak scattering from the strong direct beam it is necessary to block the direct beam and extraneous scattering from slits, *etc*., which sets a boundary for the smallest detected angle. In order to probe longer length scales with X-rays, a Bonse–Hart camera is typically used, which can access a wavevector (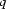) range of 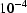
 Å^−1^ to 0.1 Å^−1^ (Diat *et al.*, 1995 ▶; Ilavsky *et al.*, 2002 ▶; Narayanan *et al.*, 2001 ▶). A conventional Bonse–Hart camera is one-dimensional collimated which is not suitable for anisotropic samples. At a cost of reduced scattering intensity, there are several papers describing a two-dimensional-collimated Bonse–Hart camera (Bonse & Hart, 1966 ▶; Konishi *et al.*, 1997 ▶; Ilavsky *et al.*, 2009 ▶). They demand a de-convolution procedure and are inefficient in comparison with the area-detector-based method available for larger wavevectors. The scanning procedure also makes it difficult for time-resolved measurements. In addition, previous works (Ehrburger-Dolle *et al.*, 2001 ▶; Shinohara *et al.*, 2007 ▶) show that ultra-small angles could be achieved by using a very long sample-to-detector distance or a very small beam stop. In this case, additional interpolations with SAXS data are required because of the limitation of the field of view. The recently introduced coherence-based X-ray near-field speckle (XNFS) technique potentially offers an improved means of studying characteristically large length scale structures with X-rays (Cerbino *et al.*, 2008 ▶). In addition, XNFS is able to extend X-ray measurements to wavevectors (length scales) at least an order of magnitude smaller (larger) than may be achieved using a Bonse–Hart camera.

The principle of XNFS is as follows. When coherent or partially coherent radiation impinges on a disordered material consisting of a number of scatterers at random locations, a random set of phase shifts will be induced on the scattering beam. As a consequence, a grainy pattern will be observed in the scattered beam a certain distance away from the material. This pattern is called a speckle pattern (Sutton *et al.*, 1991 ▶). In the near-field region, under conditions where the scattered radiation and the transmitted beam simultaneously impinge upon an X-ray area detector, high-quality speckles can also be observed resulting from coherent interference between the incident and scattered beams. This speckle is called X-ray near-field speckle (XNFS) in analogy to the near-field speckle (NFS) that was initially exploited using laser sources (Giglio *et al.*, 2000 ▶).

If, instead of a laser, one uses a high-brilliance X-ray source, it then becomes possible to study dense, optically turbid and/or absorbing media, in a range of length scales where no other X-ray or optical methods are applicable. To date there exists a single manuscript describing the extension of NFS into the X-ray regime, by Cerbino *et al.* (2008 ▶). They showed that the spatial power spectrum of XNFS is in principle simply and directly related to the sample’s structure factor [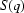] in the range of wavevectors from 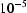
 Å^−1^ or less to 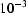
 Å^−1^ or larger. Equivalently, XNFS measures the density–density correlation function [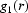] from length scales of 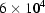
 Å or more to 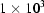
 Å or less. In addition, the evolution of the heterodyne speckle pattern in time determines the sample’s intermediate scattering function [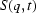] and its spatial Fourier transform, 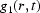. Here, we will demonstrate the implementation of XNFS measurements at beamline 8-ID-I at the Advanced Photon Source (APS) at Argonne National Laboratory, and will explore and discuss the utility and drawbacks of this method for studies of colloidal suspensions.

## 2. Basic theory

In this section we present a derivation of what we may expect to measure in XNFS experiments. We envision a sample with density 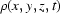 and a detector located in the plane *z* = 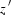, so that 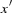 and 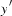 specify a given detector pixel. Then, we may write for the amplitude scattered by volume element 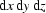 at 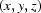 to the detector pixel at 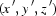 at time 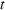,
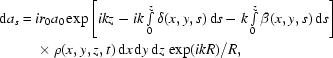
where 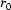 is the Thomson radius, 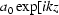 − 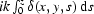 − 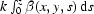] is the amplitude of the incident wave at *z*, and 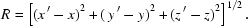

            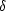 and 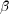 are the real and imaginary parts of the X-ray refractive index, respectively.

For a sufficiently uniform sample, for which the 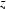-integrals of 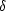 and 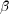 are independent of 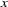 and 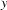, and ignoring the phase part, we may write 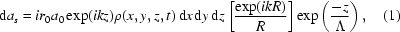
where 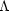 is the X-ray absorption length. Equation (1) represents a quite different regime than that used to interpret X-ray imaging experiments. Such imaging experiments instead rely on the 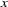 and 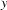 dependence of 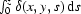 and 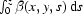 to create an image of the sample. Henceforth, we will neglect explicit mention of absorption, but otherwise take equation (1) as our expression for the scattered amplitude.

In the near-field regime, in which scattered X-rays interfere with the incident beam within the coherence area of the incident beam, we are necessarily concerned with values of 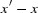 and 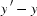 that are of the order of 100 µm or less, and values of 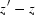 that are of the order of several millimeters or more, so that 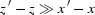 and 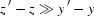. It follows that 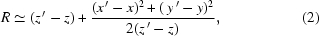
and, therefore, 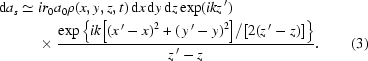
To determine the total amplitude scattered to (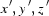), it is simply necessary to integrate over the volume (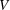) of the sample, *i.e.* 
            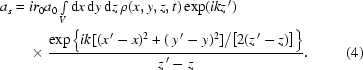
Heterodyne near-field speckle involves interference between the scattered beam and the incident beam of amplitude 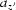. Thus, the intensity at time 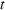 recorded at (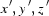) is 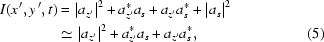
where at the detector 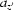 = 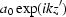 and we have taken 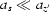.

Therefore, the measured intensity is 
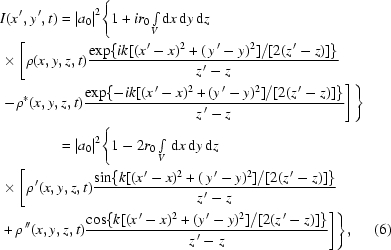
where 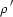 and 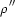 are the real and imaginary (absorptive) parts of the electron density, respectively.

Equation (6) implicitly assumes perfect transverse coherence. To incorporate the effect of a finite transverse coherence length, it is necessary to introduce a mutual coherence function, 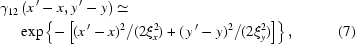
where 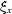 and 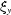 are the transverse coherence lengths in the *x*- and *y*-directions, respectively. Incorporating the effect of partial coherence, equation (6) becomes
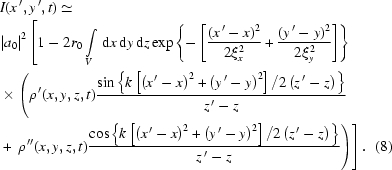
The first term of (8) is constant and the second term of (8) is a convolution in terms of real-space variables. Therefore, in Fourier space, the first term becomes a 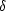-function at the origin, while the second term becomes a product. Therefore, in terms of the Fourier transform variables 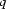 and 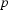 (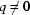 and 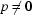), in the realistic case that the 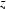-variations in the sample density occur on length scales less than 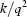, which is typically hundreds of micrometers or more, it may further be shown that the Fourier-transformed intensity is given by
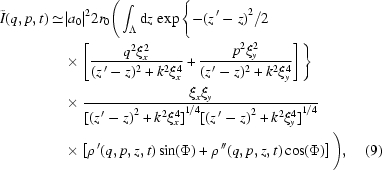
where 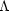 is the thickness of the sample, 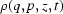 is mixed in real and reciprocal space and the phase factor 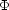 is equal to
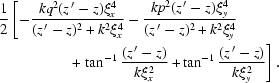
Note that the 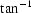 terms in the phase factor describe an on-axis phase jump of a focused beam (Gouy effect) (Gouy, 1890 ▶) and underline the well known fact that field correlations propagate as the radiation field does. So these terms account for the change in the phase in the interference between the scattered beam and transmitted beam.

**Figure 1:**
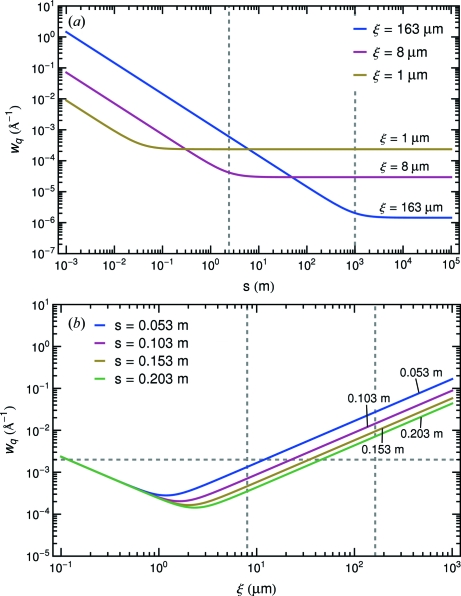
The FWHM 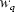 of the Gaussian distribution for the effect of partial coherence of the source beam, plotted *versus* (*a*) sample-to-detector distance for different coherence length and (*b*) coherence length for different sample-to-detector distances. In (*a*) dashed lines denote the boundaries separating far-field and near-field for 8 µm and 163 µm from left to right. In (*b*) the intersections of dashed lines and colored lines denote the differences of 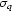 at different 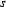 for 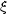 = 8 µm and 163 µm.

Equation (9), which stands as a one-dimensional convolution, may be further simplified by the following argument: the 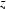-variations in 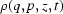 occur on length scales set by the sample’s structure, namely of the order of tens of micrometers or less. On the other hand, the 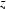-variations in the remainder of the integrand occur on a length scale given by 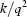, which is typically many hundreds of micrometers or more. Therefore, in the integrand it is permissible to replace each 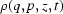 by its mean value, *i.e.* by its zero Fourier component divided by the sample thickness, *i.e.* 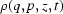 ≃ 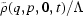. This factor, which is now independent of 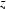, may be then taken outside of the integral, yielding
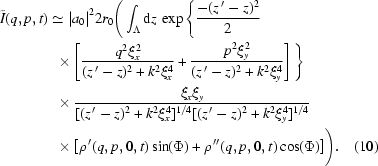
Changing variable 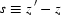, equation (10) becomes
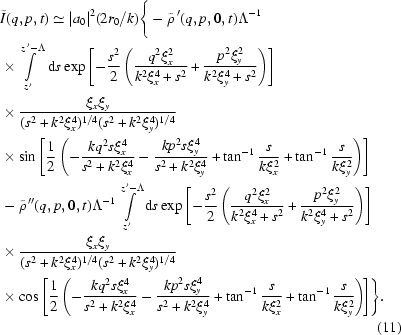
The sample thickness 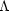 is about the diameter of the capillary equal to 0.7 mm, which is much smaller than the sample-to-detector distance 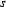 ranging from 53 mm to 203 mm. Thus, we assume that the integrand varies negligibly within the range of 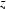 of the sample. As a result, equation (11) can be further simplified as
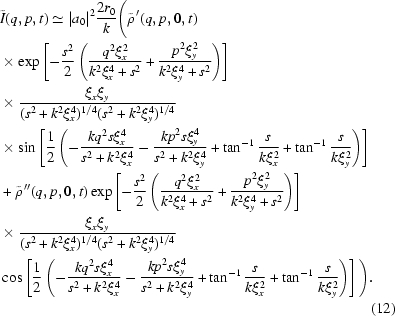
Here the term 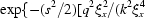 + 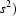 + 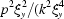 + 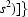 describes the effect of the partial coherence of the source beam on the speckle intensity. It is a product of two Gaussians with variances 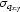 = 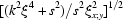. The full width at half-maximum (FWHM) of the Gaussian distribution is 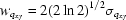. Fig. 1 ▶ plots the FWHM of coherence of the source beam *versus* (*a*) sample-to-detector distance 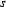 and (*b*) coherence length 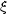. From Fig. 1(*a*) ▶ we observe that 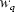 decreases as 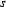 increases until it reaches a constant value at a certain distance,
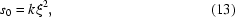
which is the usual near-field condition, called the Fresnel condition. However, for XNFS that requires that the way scattered radiation falls onto the sensor duplicates the actual angular distribution of the scattered intensity, a much stronger condition should be satisfied (Giglio *et al.*, 2000 ▶),
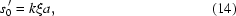
where 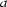 is the size of the scattering particle. With this condition the source beam fills the whole field of view and the speckle size is related to the actual size of the probing material. In Fig. 1(*b*) ▶, 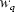 is plotted *versus* coherence length for different values of 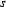. For the estimated coherence lengths at 8ID indicated *via* the dashed lines in (*b*), in principle, one expects to observe an 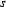-dependent change in the two-dimensional speckle intensity. However, in reality, owing to the limited spatial resolution, which in turn limits the 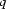 range and, more critically, the sensor response (Alaimo *et al.*, 2009 ▶), we have not been able to observe this 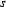-dependent variation, as we discuss in more detail below. When 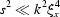 and 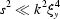 as we expect at 8-ID, then equation (12) may be simplified even further,
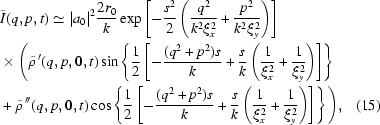
where the term 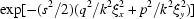 is introduced as the spatial coherence transfer function by Cerbino *et al.* (2008 ▶).

Introducing 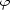 = 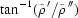, which is always small, except at X-ray energies near an absorption edge, and 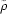 = 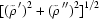, we may re-write (15) as
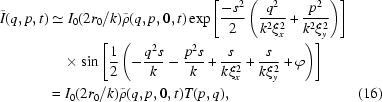
where 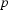 and 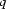 are the wavevectors obtained in the 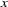
- and 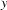-directions, respectively, by numerically Fourier transforming the CCD image, 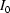 = 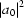, 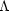 is the sample thickness, 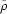 is the electron density in Fourier space, and where the latter equality defines the transfer function 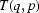. It is worth emphasizing that the transfer function 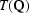 with 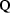 = 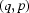 is written as
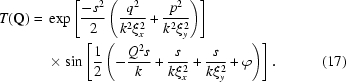
Equation (16) immediately allows us to calculate the static structure factor 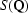 in terms of measured quantities. Specifically, 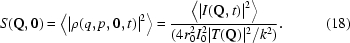
Similarly, it is straightforward to show that, in the context of XNFS, the normalized IFS is
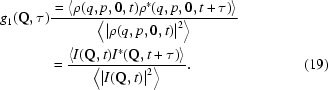
According to (19), 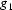 is independent of the transfer function 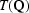, and in turn does not depend on the sample-to-detector distance 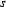.

In summary, the intensity measured in the near-field speckle experiments is proportional to the density of the sample rather than the modulus squared of the density as in conventional far-field speckle experiments like XPCS and SAXS. Thus, the time autocorrelation of 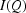 gives directly the intermediate scattering function 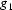 [equation (19)]. When the delay time 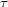 is chosen to be zero, we obtained a quantity proportional to the static structure factor 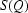 [equation (18)] times the NFS transfer function 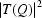.

## 3. Detector design

High spatial resolution and high detection efficiency are key goals for imaging the X-ray speckles in XNFS experiments. In XNFS experiments, in order to resolve micrometer-sized particles, it is necessary to employ detectors capable of resolving on the micrometer scale. A typical X-ray imaging detector consists of a crystal X-ray scintillator, a microscope objective and a fast high-resolution large-dynamic-range CCD-based camera. The scintillator converts X-rays into visible light; the objective collects the visible light and magnifies the visible-light image; and finally the CCD camera records the image. Our aim was to design a detector with the best combination of scintillator and objective to achieve the optimal combination of spatial resolution and detection efficiency for XNFS experiments.

A key characteristic of an objective is its numerical aperture (NA). The NA of an objective defines the largest angle of light acceptance as well as the light-collecting power. The detection efficiency scales as (NA)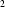. Thus, a large NA objective is necessary for high detection efficiency. It is common to use immersion oil of high refractive index (*n* = 1.515) between the front lens of the objective and the scintillator to achieve a high NA. We employed a Nikon Plan Fluor 40× oil immersion microscope objective with NA = 1.3, a working distance of 0.2 mm and a field of view of diameter 0.67 mm. This objective uses an infinity-focused optical system with a reference focal length of 200 mm. In our case, with the implementation of a tube lens with adjustable focal length from 25 mm to 150 mm, the 40× objective gives a real magnification of 5–30 times.

**Figure 2:**
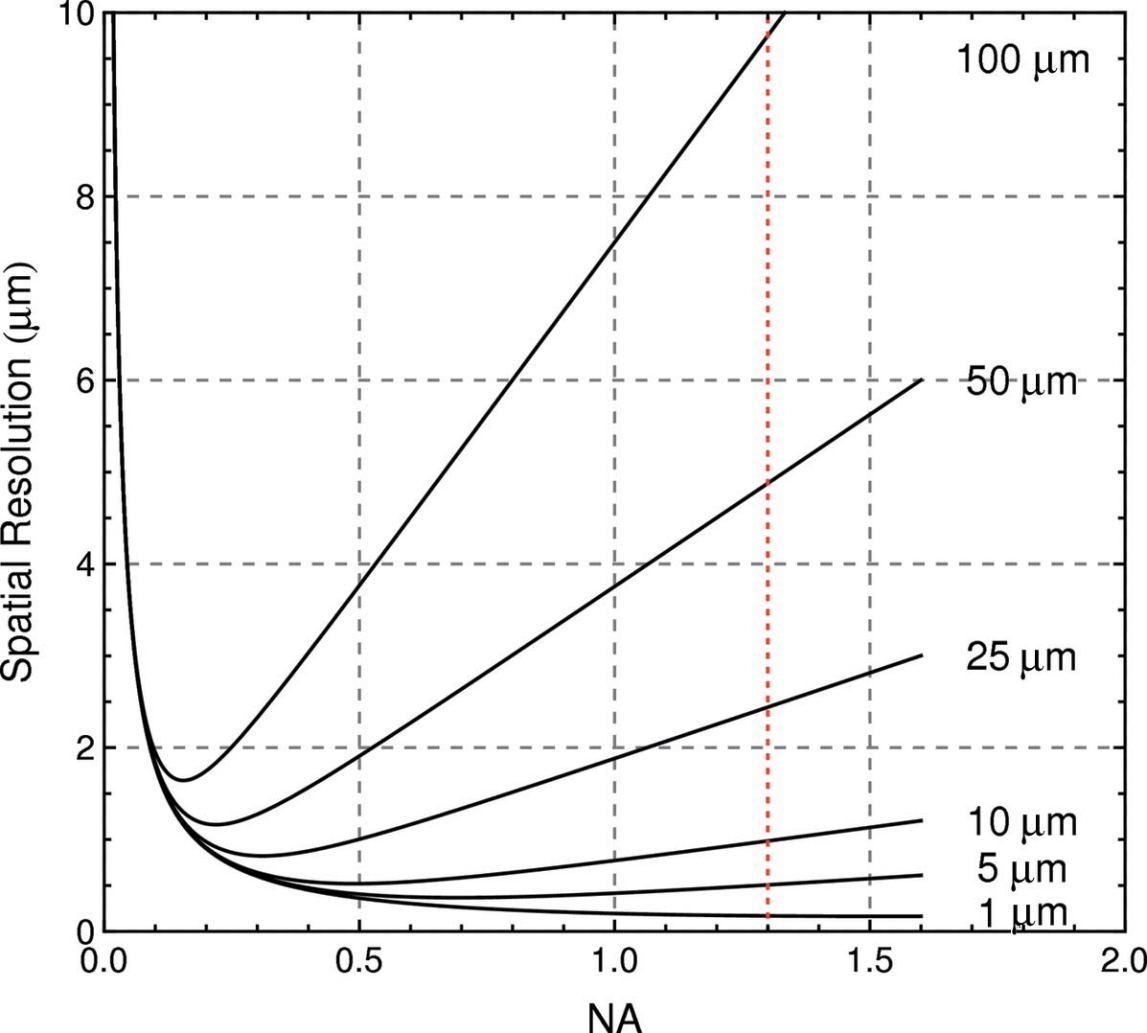
Spatial resolution *versus* numerical aperture NA of the objective for different X-ray absorption lengths of the scintillator, adapted from Martin & Koch (2006 ▶).

Generally, taking into account both the effects of diffraction and depth of focus, the spatial resolution [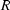] as a function of NA is given by (Martin & Koch, 2006 ▶)
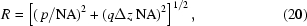
where 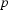 = 0.18 µm and 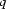 = 0.075 µm are constants obtained by numerical simulations (Martin & Koch, 2006 ▶) and 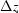 is the X-ray absorption length of the scintillator. Based on (20), one can plot 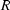 *versus* NA for different 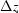, as shown in Fig. 2 ▶. It is clear from Fig. 2 ▶ that, in order to achieve a spatial resolution of a micrometer or less with high detection efficiency (NA 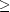 1.0), we have to choose a scintillator with an X-ray absorption length of 10 µm or less.

Besides the X-ray absorption length, several additional characteristics of the crystal scintillator are critical for X-ray imaging, including high X-ray stopping power; high light yield; the emission wavelength being compatible with CCD readout (400 nm–700 nm); a similar refractive index to the immersion oil (*n* = 1.515); and a small thickness to minimize spherical aberration. At a minimum, the scintillator thickness should be smaller than the working distance of the objective (0.2 mm), so that the objective can focus to the upstream side of the scintillator.

As shown in Table 1 ▶, one potential candidate is YAG:Ce (Y
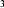
Al
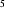
O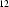:Ce), which has been widely used in X-ray imaging detectors. However, its X-ray absorption length at 7.44 keV is about 25 µm, which is not suitable for a submicrometer resolution detector with a NA = 1.3 objective (Fig. 2 ▶). Another candidate is CdWO_4_, whose X-ray absorption length at 7.44 keV is only 6.5 µm. However, it is very difficult to obtain thin crystals of CdWO_4_. It easily breaks before being thinned to the desired thickness (<0.2 mm) owing to its (010) cleavage plane. In addition, the CdWO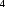 refractive index (*n* = 2.2) is much different from that of immersion oil (*n* = 1.515), which will induce a relatively large spherical abberation. In our set-up, we use LYSO (Lu_1.8_Y_0.2_SiO_5_) which appears to be the most appropriate candidate overall. Its X-ray absorption length at 7.44 keV is 9.5 µm, slightly larger than the X-ray absorption length of CdWO_4_ but still good enough to produce high spatial resolution. Its light yield is 32 photons per keV, much higher than both CdWO_4_ and YAG:Ce. Its refractive index (*n* = 1.81) is not too far from that of immersion oil inducing less spherical abberation than CdWO_4_. In addition, we found a manufacturer providing two-sided polished LYSOs with a thickness of 0.15 mm, just appropriate for our objective, although even thinner would lead to reduced spherical aberrations (Koch *et al.*, 1998 ▶; Born & Wolf, 1999 ▶).

## 4. Experimental set-up

**Figure 3:**
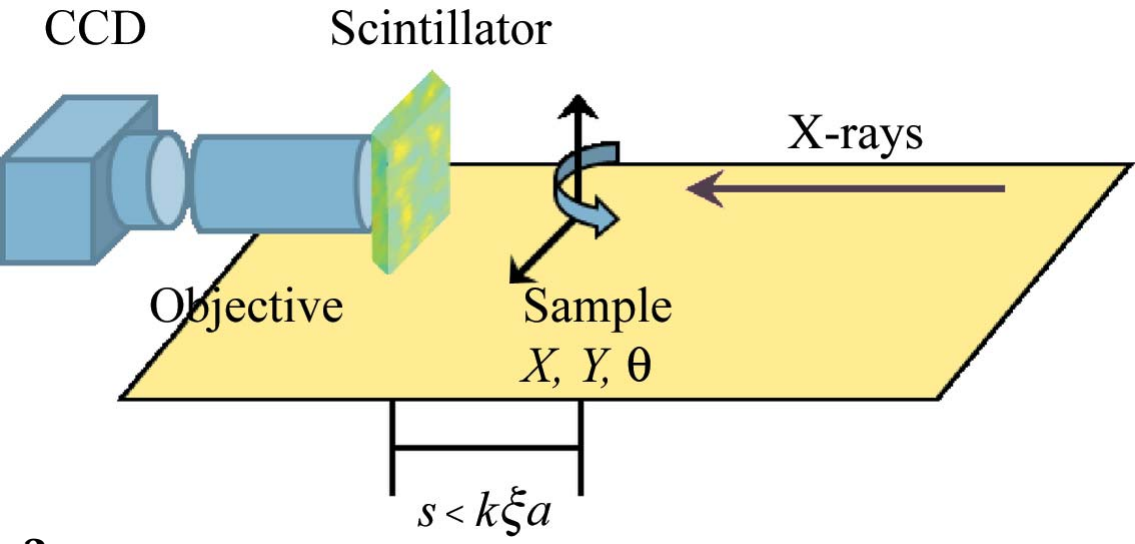
Sketch of the optical set-up for XNFS experiments. The key components from right to left are: beam source, sample stage, scintillator, microscope objective and CCD camera.

XNFS measurements were carried out at beamline 8-ID-I of the APS at Argonne National Laboratory, using X-rays of energy 7.44 keV and half the source beam size available at the 8-ID-I hutch, about 0.5 mm × 0.5 mm. Fig. 3 ▶ shows a sketch of the optical set-up located in the 8-ID-I hutch. From right to left we have the beam source, the sample stage, the scintillator, the microscope objective and the CCD camera. In XNFS experiments, no spatial and spectral filtering of the direct beam are required (Cerbino *et al.*, 2008 ▶). We inherit the exiting set-up for XPCS which gives an energy resolution of 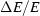 ≃ 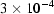 and remove all the slits letting the full beam impinge onto the sample, and record the interference pattern of the transmitted and scattered beams by means of our detector placed at distances from the sample ranging from 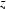 = 53 mm to 203 mm.

As described before, we use two-sided polished LYSO (Lu_1.8_Y_0.2_SiO_5_) with a thickness of 0.15 mm to convert X-rays into visible light and a Nikon Plan Fluor 40× oil immersion microscope objective with a numerical aperture of NA = 1.3 to magnify the image. Both of them are mounted on a piezo-electric stage which has a mechanical manual adjustable coarse travel range of 4 mm and a piezo-electric travel range of 20 µm with a resolution of 20 nm. As a result, by carefully tuning the distance between the objective and the scintillator, we are able to focus the image which is in turn magnified 5 to 30 times by a tube lens with adjustable focal length and then recorded by a ‘CoolSNAP’ CCD camera made by Photometrics. The camera features 1392 × 1040 pixels of size 6.45 µm × 6.45 µm and a maximum frame rate of 56 Hz. All the images so-obtained are subsequently cropped to 1024 × 1024 pixels for convenience of two-dimensional Fourier transformations in the data analysis.

There are several contributions to the detection resolution.

(i) The resolution of the scintillator, for X-rays incident onto the scintillator at a single point. According to Koch *et al.* (1998 ▶) and Martin & Koch (2006 ▶), this is typically 0.1 µm for 7–8 keV X-rays, smaller than optical limits on the resolution.

(ii) The resolution determined by the diffraction limit and the defect of focus of the objective and the scintillator *via* equation (3). With an objective with NA = 1.3 and LYSO with an X-ray absorption length of 9.5 µm, we obtain a spatial resolution of 0.98 µm.

(iii) The reduction in resolution caused by the spherical aberration due to the use of a scintillator of mismatched refractive index, which is proportional to the thickness of the scintillator and the cube of NA (Koch *et al.*, 1998 ▶; Born & Wolf, 1999 ▶). Since our objective is corrected for spherical aberration and the additional spherical aberration induced by replacing the glass coverslip by the scintillator can be compensated for by adjusting the oil thickness, this factor is not critical to the spatial resolution.

(iv) The (demagnified) size of the CCD pixel, if too large, could limit the resolution. For a magnification of 30, the CoolSNAP can resolve a length scale as small as 6.45/30 = 0.215 µm which does not limit the resolution. As a result, the spatial resolution of the detector should be largely determined by factor (ii), which is about 1 µm.

Hence, we are able to estimate the maximum 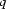 range. With 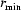 = 0.98 µm, in reciprocal space, 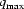 = 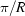 ≃ 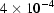
 Å^−1^. On the other hand, the lower limit of the wavevector 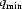 should be determined by the largest accessible length scale. In principle, the largest length scale is the size of the measured scattering image equal to 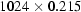
 µm = 0.22 mm, so 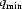 = 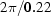
 mm ≃ 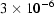
 Å^−1^. However, practically our data show an identical low 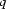 profile that is independent of which sample is being studied and of the sample-to-detector distance. This is likely to be due to the beam structure on long length scales. So, the realistic useful 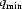 is of the order of 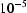
 Å^−1^. Nevertheless, the 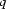-range achieved is at least a decade below the range accessible to the conventional XPCS experiments.

## 5. Silica 0.45 µm

The first sample we measured at 8-ID-I, as an initial test of our XNFS set-up, was a colloidal suspension of silica particles of diameter 0.45 µm and a volume fraction (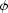) around 0.05 in water. The sample is injected into a 0.7 mm-diameter boron glass capillary for X-ray measurements with an energy of 7.44 keV and a flux density of 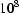
 s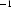. The static structure factor peak of this sample is expected to be located around 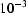
 Å^−1^, which means that the sample has a uniform scattering profile in the 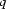 range accessible in our XNFS set-up [*i.e.* 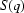 ≃ a constant]. In other words, the intensity profile 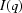 we measured from this silica suspension should simply result from the transfer function 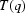 [equation (16)]. Thus, it should be an ideal sample to examine the sample-to-detector distance (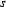) dependence of 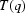.

**Figure 4:**
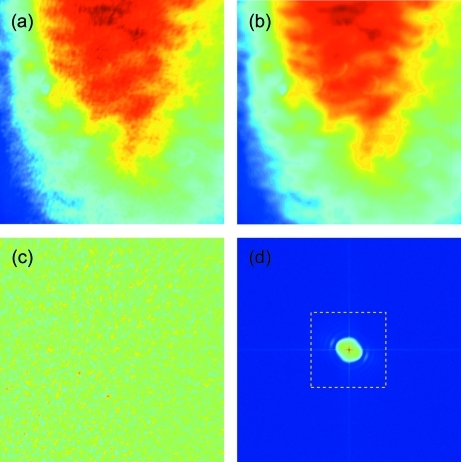
(*a*) A raw single frame of a scattering image of SiO_2_ of diameter 0.45 µm at a sample-to-detector distance of 53 mm. (*b*) The image averaged over 1000 frames. (*c*) Normalized image obtained by dividing the raw image by the averaged image. Each image consists of 1024 × 1024 pixels. (*d*) Two-dimensional Fourier transform of (*c*).

Illustrated in Fig. 4(*a*) ▶ is a typical raw image of the silica suspension measured at 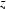 = 53 mm. The image contains 1024 × 1024 pixels with a pixel size of 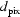 = 6.45 µm. The magnification of the detector is set to 30. Thus this image corresponds to a region of size 0.22 mm × 0.22 mm in the sample. The speckle pattern appears quite obscure and weak owing to the large-scale static background. After averaging over 1000 frames, the speckle pattern is washed away leaving the static beam profile fluctuation unchanged, as shown in Fig. 4(*b*) ▶. In order to remove the large-scale static fluctuations, we perform a normalization of each raw image by dividing each one with an average image, averaged over 1000 frames, as shown in Fig. 4(*b*) ▶. Fig. 4(*c*) ▶ presents one example of the resultant image, which reveals a clear uniform speckle pattern [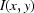]. Next, a two-dimensional discrete Fourier transform is performed *via* 
            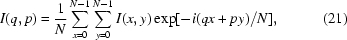
which produces the 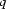-space image, as shown in Fig. 4(*d*) ▶. Here, *N* = 1024. As a result, the corresponding 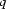 coordinates are given by
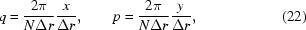
where 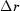 is the size of one pixel, equal to 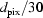 = 0.215 µm.

**Figure 5:**
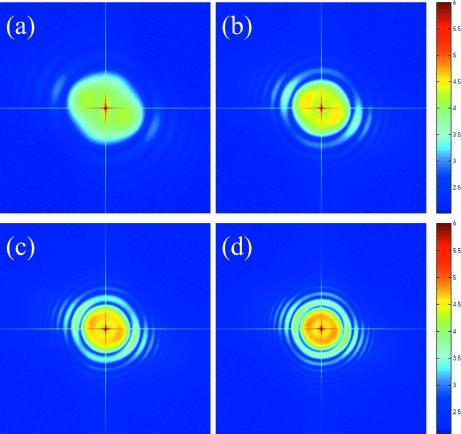
Enlarged Fourier-transformed scattering image of the SiO_2_ suspension at different sample-to-detector distances (*a*) 53 mm, (*b*) 103 mm, (*c*) 153 mm and (*d*) 203 mm.

We report in Fig. 5 ▶ several examples of the magnified Fourier-transformed image corresponding to the region inside the dashed lines in Fig. 4(*d*) ▶. Different panels are obtained at different sample-to-detector distances: (*a*) 53 mm, (*b*) 103 mm, (*c*) 153 mm and (*d*) 203 mm. In each image, prominant fringes can be seen. It is clear that the fringes become finer when the detector is moved away from the sample. This agrees with the theoretical prediction that the transfer function 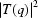 is proportional to a sine term whose frequency depends on the sample-to-detector distance [equation (17)]. Note that the rings of the Fourier transformation are not azimuthally uniform. This effect may be related to asymmetry in the coherence of the source beam. However, the envelope of the asymmetry has no obvious 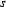-dependence, in contrast to what may be expected on the basis of equation (12). Thus, we do not understand this asymmetry in detail. In fact, examination of these data (Fig. 5 ▶), in the light of equation (12), suggests that the predicted effect of a finite coherence length is not playing a role in determining these data, presumably because the width of the Gaussian in equation (12) is greater than our accessible 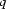-range of 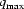 ≃ 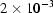
 Å^−1^, even for the largest values of 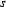 studied. Alaimo *et al.* (2009 ▶) showed that by far the largest contribution to the 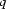 decay of the speckle power spectrum is due to the sensor transfer function. It is highly possible that these asymmetry rings are due to the sensor response. This is supported by the slightly elongated speckles in the direction orthogonal to that of the power spectra, as shown Fig. 4(*c*) ▶. Ideally, an accurate sensor transfer function should be obtained when the sample is placed close enough to the sensor. However, owing to the limitation of our set-up, 53 mm is almost the closest distance we can reach.

**Figure 6:**
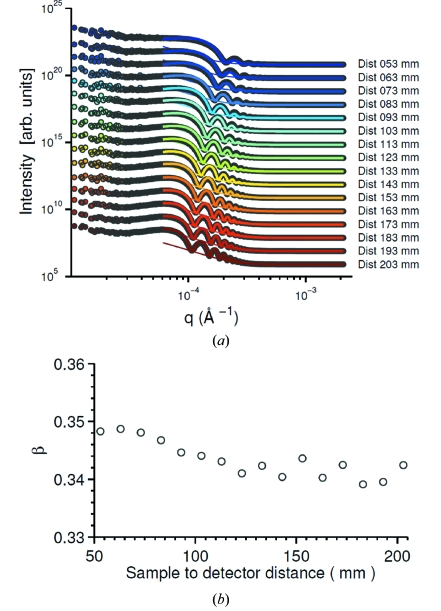
(*a*) Intensities of the SiO_2_ suspension at different sample-to-detector distances. Symbols are the data. Thick solid lines are the fittings based on equation (23). Thin solid lines are obtained by fitting the local minima with a stretched exponent, describing the contribution of multiple scattering. Data are displaced by a factor of ten for clarity. (*b*) The best-fit exponent 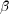 *versus* sample-to-detector distances varying from 53 mm to 203 mm for the silica 0.45 µm suspension.

To quantify 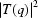, we plot in Fig. 6(*a*) ▶ the azimuthally averaged intensity profile (symbols) *versus* wavevector (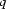) for different values of 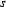 varying from 203 mm to 53 mm with a decrement of 10 mm. The thick solid lines in Fig. 6(*a*) ▶ are the fits of 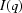 to the relation
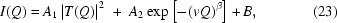
with *Q* = 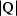 = 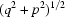 and 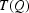 as a simplification of (17),
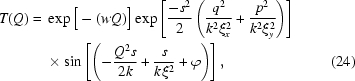
which consists of a product of three terms. The first factor is additional to equation (17) in order to take into account the contributions from the sensor transfer function (Alaimo *et al.*, 2009 ▶); the second term derives from the partial coherence of the incident beam. How the coherence length enters is somewhat counterintuitive: it is proportional to the width of the Gaussian term in reciprocal space. However, this term is set to unity for fitting, since it plays no significant role in the accessible 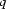 range, as we observed in the context of Fig. 5 ▶. The third term is a sine function that describes the fringes produced by the interference of the scattered beam and incident beam. In the X-ray domain, this term is called the phase-contrast transfer function, and is related to the so-called Talbot effect in imaging measurements (Cerbino *et al.*, 2008 ▶). Note that we assume 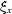 = 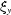 = 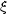 for the simplification of a phase factor in the sine function.

**Figure 7:**
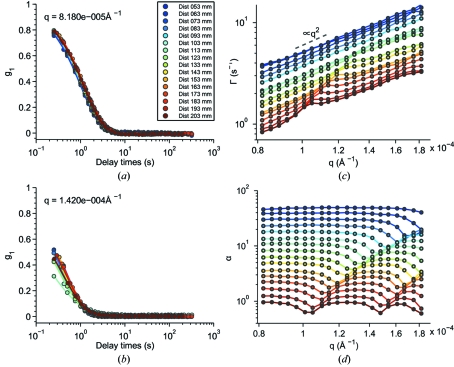
Normalized intermediate scattering functions of the SiO_2_ suspension *versus* delay time 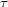 measured at different sample-to-detector distances with (*a*) *q* = 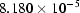
 Å and (*b*) *q* = 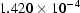
 Å^−1^. (*c*) The best-fit decay rate 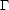 *versus* wavevector 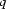. Points are the fitting results and the lines are guides to the eye. Data are displaced by a factor of 1.1 for clarity. (*d*) The best-fit exponent 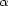 *versus* wavevector 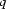. Data are displaced by a factor of 1.3 for clarity.

Besides the contribution of 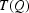 and background noise, the second term in (23), in the form of a stretched exponential decay, describes another experimentally significant contribution to the intensity profile: namely multiple scattering. The existence of multiple scattering is evident based on three observations. Firstly, the sine-squared term goes to zero periodically, which should make the intensity profile exhibit minima with the same magnitude. However, the measured minima decrease with 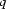. The second piece of evidence pointing to the importance of multiple scattering comes from the dynamic data (see later), which display a 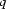-dependent decay rate and exponent that mirrors and anti-mirrors the form of 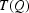 [Figs. 7(*c*) and 7(*d*) ▶]. This indicates that we are measuring faster dynamics owing to multiple scattering where single scattering vanishes. Thirdly, very strongly scattering samples, *i.e.* deliberately multiply-scattering samples, show no minima at all.

Hence, we fit the intensity data in two steps. In the first step we focus on multiple scattering. Note that the single-scattering term vanishes at values of 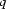 satisfying 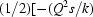 + 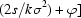 = 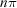, where *n* = 1, 2, 3,…. As a consequence, we extract the intensity at the 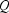 values corresponding to those minima in 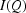 and fit them with only the multiple-scattering term plus the background. The characteristic length 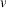 is fixed to 500 µm. The amplitude 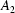 and the exponent 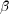 were allowed to vary. The resultant multiple-scattering term is plotted as the thin solid lines in Fig. 6(*a*) ▶. The best-fit exponents 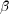 are plotted in Fig. 6(*b*) ▶ *versus* the sample-to-detector distance 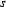. 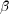 fluctuates around 0.345, giving an empirical stretched-exponential form for the intensity of the multiple scattering. Next, the remaining intensities after subtracting the filled multiple scattering and background are fitted with the transfer function [equation (24)]. We use the measured sample-to-detector distance 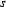 and try to find one set of 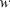, 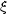 and 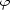 that works for all the values of 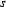. The only varying parameter is the overall amplitude. The set 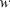 = 1.8 µm, 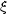 = 163 µm and 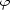 = 0.026 yields a good fit for all values of 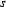 studied, as shown by the thick solid curves in Fig. 6(*a*) ▶. 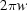, the characteristic length scale of the sensor transfer function, is about 11 µm, 17 pixels on the detector (Cerbino *et al.*, 2008 ▶; Alaimo *et al.*, 2009 ▶). The small value of 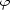 indicates that the X-ray absorption for this silica sample is essentially small. In general, the fitting reproduces the data with few fitting parameters, confirming the theoretical relation between the sample-to-detector distance 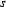 and the transfer function 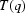, and consequently confirming the feasibility of our experimental set-up.

With the same principle of XPCS, the fluctuations of near-field speckles should reflect the dynamics of the sample. As shown in equation (19), the time autocorrelation of the intensity gives rise to 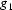 instead of 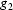 in XNFS experiments. Hence, we have presented in Fig. 7(*a*) ▶ the normalized intermediate scattering function [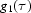] *versus* delay time (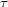) for 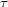 between 0.4 s and 319 s corresponding to an exposure time of 0.2 s for different 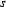 at 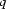 = 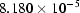
 Å^−1^. The values of 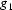 collapse into one curve for different 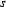, which agrees with the theoretical prediction that 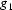 has no 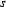-dependence owing to the cancelation of 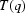. However, for a larger wavevector of *q* = 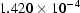
 Å^−1^, the 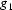 values do not overlap for different 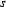, as shown in Fig. 7(*b*) ▶. To elucidate the reason for this discrepancy and quantify our observations, we have fitted 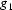 measured at different 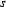 and 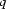 to a stretched exponential form,
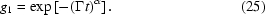
The best-fit relaxation rate (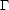) [equation (25)] *versus* wavevector 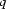 is illustrated in Fig. 7(*c*) ▶. The values of 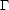 at successive 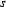 are displaced by a factor of 1.1 from the previous 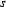 value for clarity. Generally, 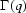 at different 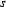 show a 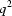 behavior, illustrated by the dashed line in Fig. 7(*c*) ▶. However, peaks are observed at the 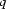 positions coinciding with the 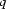 positions of the dips in the transfer function 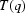 (Fig. 6*a* ▶).

Away from these multiple-scattering peaks, 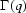 increases as 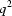 *versus* 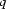, which is reasonable for a SiO_2_ suspension undergoing Brownian motion. Quantitatively, for Brownian motion, we expect 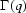 = 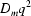. As a result, we derive the value of the diffusion coefficient 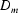 = 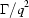 ≃ 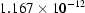
 m^2^ s^−1^. According to the first-order hydrodynamic interactions, 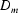 = 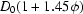 (Batchelor, 1976 ▶), where the Stokes–Einstein diffusion coefficient 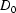 = 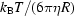 ≃ 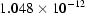
 m^2^ s^−1^ with 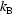 the Boltzmann constant, 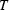 the room temperate equal to 293 K, 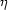 the dynamic viscosity equal to 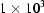
 kg m^−1^ s^−1^, we obtain 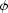 ≃ 0.07, which is reasonable.

At the 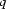 positions of the peaks, where the single-scattering amplitude goes to a minimum because of the zeros of the sine-squared term in 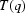, we hypothesize that we are measuring multiple scattering of the sample. This theory explains why we obtain faster dynamics at those 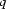 positions (Berne & Pecora, 2000 ▶). Fig. 7(*d*) ▶ shows the corresponding best-fit exponent 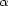, which exhibits similar fluctuation patterns as 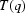 and supports our hypothesis. Underlying this hypothesis is the idea that the rapid variations of 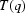 *versus* 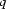 may be associated with single scattering, whereas the intensity of multiple scattering likely shows a relatively smooth 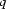-dependence. Accordingly, if, for a particular set of data, the scattering minima owing to 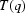 are indistinct (do not send the scattering intensity to zero), then it follows that the XNFS data set in question suffers from multiple scattering. Hence, to calculate 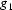, we have to pick 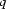 smaller than the first dip of 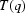 so that the measured coherent scattering is reliable.

**Figure 8:**
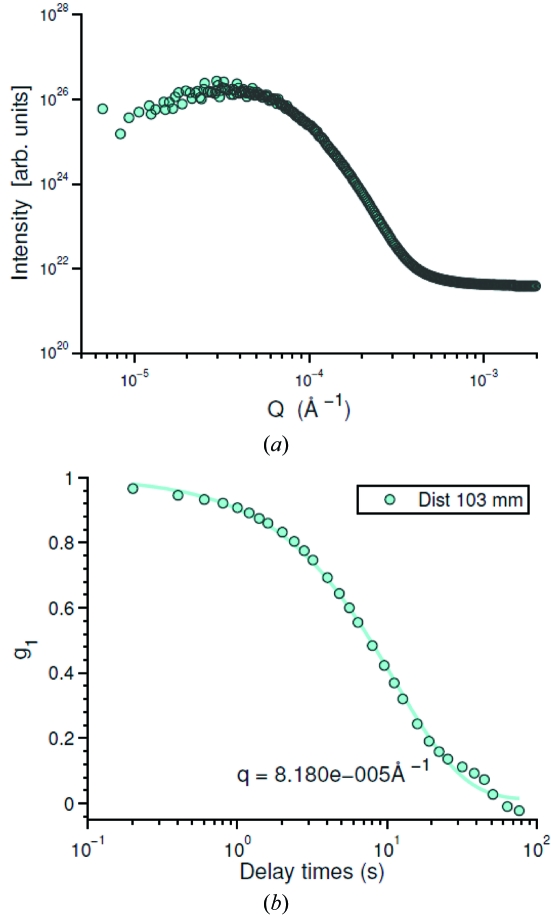
(*a*) Intensities of 3 mm-thick Gillette shaving foam at a sample-to-detector distance 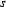 = 103 mm. (*b*) The corresponding normalized intermediate scattering functions (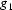). The symbols are the data. The lines are fittings based on a stretched exponential decay [equation (25)].

To further test this idea, we carried out measurements on a sample that could be expected to show very strong scattering and therefore strong multiple scattering, namely a 3 mm-thick sample of Gillette Foamy shaving foam, which is know to consist of a dense foam of micrometer-sized air bubbles in aqueous liquid. Fig. 8(*a*) ▶ shows the scattering intensity from such a sample as a function of 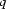, obtained using the XNFS prescription. However, in contrast to the more weakly scattering silica spheres, discussed above, evidently in this case there are not the oscillations in intensity that are expected for XNFS, *i.e.* there is no evidence that the XNFS 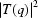 is displayed in these data. We infer that this is indeed the result of multiple scattering and that the X-ray scattering from the 3 mm-thick foam is completely in the multiple-scattering regime. This implies that 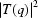 is a signature of single scattering. We can also calculate 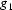 for the foam according to the XNFS prescription. This is shown in Fig. 8(*b*) ▶. The dynamics are rather slow, of the order of 0.1 s^−1^.

These results point to another difficulty with the XNFS method (which is common to ultra-small-angle X-ray scattering methods in general), namely that multiple scattering must be carefully considered and if possible eliminated. In the case of the foam, a sufficiently thin sample (much thinner than 3 mm) would have eventually reached the single-scattering regime. Interestingly, in the case of XNFS, in contrast to more traditional USAXS methods, the existence or not of multiple scattering may be straightforwardly and immediately recognized from the intensity profile, *i.e.* 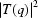, as we discussed previously.

## 6. Polystyrene 4 µm

**Figure 9:**
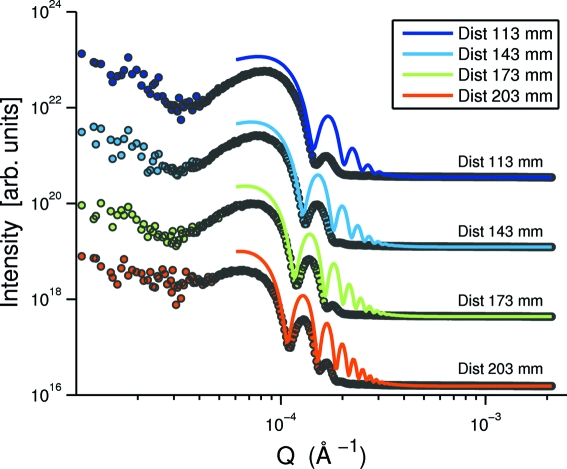
Intensities of the polystyrene 4 µm suspension at different sample-to-detector distances. The discs of different colors are the data measured at different sample-to-detector distance. The solid lines are theoretical plots of the transfer function 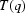 of the silica suspension for comparison. The data are displaced by a factor of 30 for clarity.

In this section we present the XNFS data obtained from a colloidal suspension of polystyrene particles of diameter 4 µm. Similar to the preparation of the silica sample, this sample is injected into the same boron glass capillary, thus resulting in a sample thickness of 0.7 mm. This sample is not as stable as the last sample, since particles with 4 µm undergo sedimentation. The static structure-factor peak of the polystyrene suspension of this size lies within the 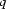-range accessible by our XNFS set-up. Hence, we expect to observe more complicated intensity profiles with the contributions from both structure of the suspension and the transfer function. Illustrated in Fig. 9 ▶ are the scattering intensities (symbols) plotted *versus* 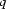 obtained by azimuthally averaging the Fourier-transformed scattering images over 1000 frames for sample-to-detector distances 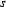 = 113 mm, 143 mm, 173 mm and 203 mm (from top to bottom). The corresponding transfer functions [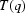] obtained by fitting data of a silica sample measured at the same 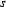 are plotted as solid lines with the same colors for easy comparisons for the peak positions of 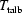. The intensity data deviate from the lines. Firstly, the peak positions of the data match those of 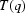. There might be one extra peak located at 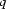 around 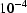
 Å^−1^ for all 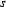, which comes from the static structure factor peak. In addition, the peaks of this sample are less sharp than those of the silica sample. This indicates that the power spectra are largely suffered from the sensor transfer function.

**Figure 10:**
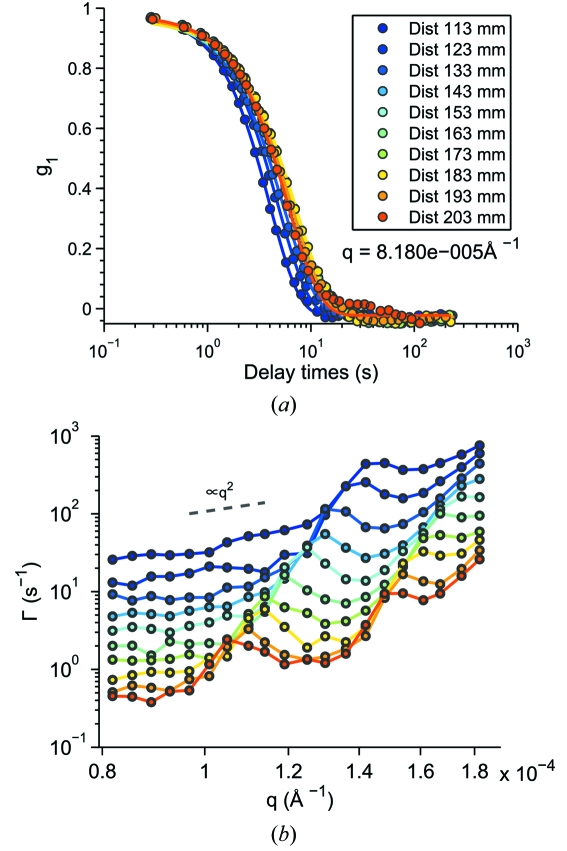
(*a*) Normalized autocorrelation functions of the polystyrene 4 µm suspension measured at different sample-to-detector distances. (*b*) The best-fit decay rate 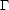 *versus* wavevector 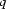. Points are the fitting results and the lines are guides to the eye. Data are displaced by a factor of 1.5 for clarity.

Illustrated in Fig. 10(*a*) ▶ are the normalized intermediate scattering functions (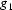) at *q* = 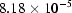
 Å^−1^ for delay times from 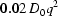 to 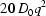
 s and sample-to-detector distances 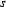 from 203 mm to 113 mm with an interval of 10 mm. The 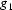 for the polystyrene suspension do not totally overlap for different 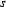 at this 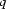 position, but decay slightly faster when the detector moves closer to the sample stage. We reason that this is the result of the sedimentation of the polystyrene particles, which leads to a denser sample with faster dynamics.

Following the same procedure as for the silica sample, the best-fit relaxation rates (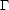), obtained by fitting one of the 100 frames 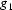 with a single exponential form [equation (25)], are plotted *versus* wavevector 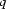 for different 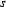 in Fig. 10(*b*) ▶. The values of 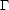 at different 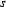 are displaced for clarity. Similarly, peaks that correspond to dips of transfer function are observed, confirming our conclusion about the measurement of multiple scattering at the minima of the transfer function. In this case the peaks are more visible than those observed in the silica sample, indicating stronger multiple scattering in this sample with larger polystyrene particles.

## 7. Future work and conclusion

In conclusion, we have presented the implementation of the new coherent X-ray technique, X-ray near-field speckle, as well as its applications and limitations. Clearly, XNFS is capable of obtaining ultra-small-angle X-ray scattering and X-ray photon correlation spectroscopy with its simple set-up and direct relationship to the density correlation function. It effectively extends to wavevectors an order of magnitude smaller than the wavevector range covered by conventional SAXS and XPCS, and enables us to explore the static and dynamic structures of micrometer-sized samples. We believe this technique will be valuable for optically dense and turbid samples which induce strong multiple scattering optically.

Technically, XNFS is not difficult to realise. A speckle pattern is produced simply by letting both scattered beam and transmitted beam impinge onto the detector. It does not require spatial filtering as it did in XPCS, which allows us to use the whole source beam and in turn greatly enhance the speckle contrast. As a consequence, it does not require laborious alignments. All the efforts were devoted to the design of the detector. A high numerical aperture objective was employed to produce high spatial resolution and efficient light collection. The measurements give convincing results, which proves the feasibility of this set-up. Improvements could be made on several aspects. One is to utilize a thinner scintillator, which will give rise to reduced spherical aberration. A faster CCD camera will improve the probing range of the dynamics of this technique.

One key difficulty of this technique is due to the transfer function 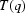. It entangles with 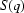. It is straightforward to characterize the structure-factor peaks and dips located at positions smaller than the 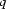 position of the first dip of 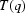. However, this would make the reliable 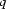 range very small. If XNFS is to realise its full potential, it will be necessary to figure out an effective way to deconvolve the static structure factor from the transfer function in the future. One possible way is to use sample-to-detector distances as small as possible, although with a cost in scattering contrast. Strong absorption samples might not be affected by this factor owing to the phase factor induced in the sine term of 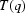 [equation (24)]. Another possible improvement might be made by measuring a control sample with exactly the same material but uniform 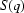 in the accessible 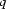 window, then dividing the intensity profile of the interested sample by that of the control sample. Another important issue is multiple scattering. Evidence of the existence of multiple scattering comes from the intensity profile and sample-to-detector dependent decay rate. Making the sample as thin as possible should solve this problem.

## Figures and Tables

**Table 1:** Characteristics of the scintillators

Scintillator	X-ray absorption length (µm)	Light yield per keV	Refractive index	Density (g cm^−3^)	Wavelength of maximum emission (nm)
LYSO (Lu_1.8_Y_0.2_SiO_5_:Ce)	9.5	32	1.81	7.1	420
YAG:Ce (Y_3_Al_5_O_12_:Ce)	25	8	1.82	4.55	550
CdWO_4_	6.5	12 to 15	2.2	7.9	475
